# Ensemble Machine Learning for Malaria Diagnosis in Resource-Limited Settings Using Clinical and Demographic Features

**DOI:** 10.3390/idr18040072

**Published:** 2026-07-13

**Authors:** Panashe Nyengera, Hilary Takunda Takawira, Farai Fredric Mlambo

**Affiliations:** 1 Department of Applied Biosciences and Biotechnology, Midlands State University, Private Bag 9055, Gweru, Zimbabwe; takawirahilary@gmail.com; 2 Graduate School of Business Administration, University of the Witwatersrand, Johannesburg 2050, South Africa; farai.mlambo@wits.ac.za

**Keywords:** malaria diagnosis, machine learning, ensemble models, resource-limited settings

## Abstract

Background: Sub-Saharan Africa suffers the greatest impact of malaria, with the 2024 Health Organization (WHO )report stating that the region represents 94% of global cases and 95% of deaths. Challenges in malaria elimination stem from weak health systems and limitations of traditional diagnostic methods like microscopy and malaria Rapid Diagnostic Tests (mRDTs), which result in missed diagnoses, delays in treatment, and preventable fatalities in resource-limited settings. This paper addresses these diagnostic limitations by developing and systematically evaluating a machine learning (ML) framework for malaria diagnosis that leverages routine clinical symptoms and demographic information tailored for these environments. Methods: Examining 637 patient records from Gutu Mission Hospital and Gweru Provincial Hospital in Zimbabwe, the research analyzed clinical symptoms (fever, chills, abdominal pain, headache, diarrhea) and demographic data (age, gender, residence, travel history). Data preprocessing involved addressing class imbalance with the Synthetic Minority Oversampling Technique (SMOTE) and employing Recursive Feature Elimination (RFE) for feature selection. Seven ML models were trained: Logistic Regression, Random Forest, Decision Trees, Gradient Boosting, K-Nearest Neighbor, Naive Bayes, and XGBoost. These individual models were used to construct ensemble models like Bagging, Stacking, Soft Voting, and AdaBoost. Performance metrics included accuracy, precision, confusion matrices, recall, F1 score, and AUC-ROC. Results: Statistically significant predictors for malaria included chills (*p* = 0.001), fever (*p* = 0.003), diarrhea (*p* = 0.01), and abdominal pain (*p* < 0.001), with travel history showing significance among demographic factors (*p* = 0.02). The stacking ensemble model yielded superior performance, achieving an accuracy of 0.96, precision of 0.95, recall of 0.98, F1 score of 0.96, and AUC-ROC of 0.98. Conclusions: This study underscores the potential of ML, particularly ensemble techniques, to enhance malaria management in resource-limited settings, providing a scalable and cost-effective diagnostic alternative that utilizes accessible clinical and demographic data, thereby supporting healthcare workers and control programs in areas where traditional methods are inadequate.

## 1. Introduction

More than half of the world population is affected by malaria, a parasitic disease that remains a major public health problem [1]. The 2024 World Health Organisation (WHO) malaria report estimates that 263 million cases with 597,000 deaths occurred in 2023. The malaria report also highlights that the WHO African regions, especially Sub-Saharan Africa, continues to shoulder the heaviest burden, contributing 94% of the cases and 95% of the deaths globally [2]. Malaria disproportionately affects the rural areas, especially poor communities with limited to no access to healthcare [3,4]. Annually, over five million people are at risk of contracting malaria in Zimbabwe. Zimbabwe malaria statistical reports indicated 16794 cases and 32 deaths in the first half of 2024 [5]. Of those cases, 199 were children under five years of age, an indication of the persistent burden of malaria within the country. Many at-risk groups continue to miss out on needed services to prevent, diagnose and treat malaria [2].

Malaria is an infectious disease caused by the Plasmodium parasite [6,7]. The most severe malaria cases are caused by the Plasmodium falciparum species [7,8]. Traditional parasitology diagnostic methods, such as malaria Rapid Diagnostic Tests (mRDTs) and microscopy, are widely used for malaria diagnosis [9]. Microscopy is the gold standard for malaria diagnosis since the early 20th century, but it is time-intensive, requires skilled personnel, and is prone to variability in accuracy depending on operator expertise [10,11]. Similarly, mRDTs are among the most efficient tools for accurately determining a patient’s malaria status. Still, their sensitivity is reduced at low parasite densities, producing false-negative or false-positive results [11,12]. Resource-limited settings are usually affected by the absence of a definitive diagnosis, which is a serious obstacle to treatment compliance, efficacy, and clinical care of severe malaria cases [8,13].

In addition to diagnostic challenges, progress towards elimination is hindered by the growing resistance to antimalarial drugs and insecticides used in vector control strategies [14]. Mosquito vectors have developed resistance to pyrethroids used in insecticide-treated nets and indoor residual spraying [15]. The rising burden of P. falciparum resistance to anti-malarial drugs such as chloroquine and sulfadoxine-pyrimethamine have been widely documented in Kenya, Tanzania, Sudan, Malawi, and Nigeria, leading to artemisinin-based combination therapy (ACTs) [16,17]. ACTs are currently used for therapeutic management for malaria, and are recommended as the first-line treatment for uncomplicated P falciparum [17]. Vaccines such as RTS, S/AS01 (Mosquirix), R21/Matrix M have demonstrated promising efficacy and are currently being used in efforts to control malaria [18].

Traditional statistical methods, such as logistic regression (LR) and time series analysis, have been used in malaria research to identify risk factors and model malaria prevalence [19]. However, they have been found to depend on data assumptions, focus on explanation over prediction, and struggle with high-dimensional clinical data, but are very useful in understanding malaria trends [19,20]. Machine Learning (ML), a subfield of Artificial Intelligence (AI) that focuses on developing the algorithms that can learn patterns and relationships from data without being explicitly programmed, offers an alternative to supporting malaria diagnosis in resource-limited settings [21,22,23].

A study focusing on ML for malaria prediction using clinical and demographic features was carried out in Uganda [3]. Individual classifiers such as Random Forest (RF), Support Vector Machines (SVM), Gradient Boosting (GB), Decision Trees (DT), Naive Bayes (NB) and K-Nearest Neighbours (KNN) were used to build models using the clinical and demographic features [3]. The individual ML model performance was evaluated using metrics such as accuracy, precision and recall, and they all achieved a good performance [3]. Ensemble models such as bagging, AdaBoost, soft and hard voting and stacking were created from the individual models. The ensemble models outperformed the individual classifiers, achieving over 0.98 across the same metrics [3].

In Yumman Province, China, a study compared the performance of traditional time series models and deep learning algorithms for malaria prediction [8]. The autoregressive integrated moving average (ARIMA), seasonal and trend decomposition using Loess (STL+ARIMA), back propagation artificial neural network (BP-ANN), and long short-term memory (LSTM) network models were applied separately in simulations using malaria data and meteorological data [8]. GB regression trees were used to combine the four models (ARIMA, STL+ARIMA, BP-ANN and LSTM) through stacking [8]. The findings reported that predictive performance of the stacking ensemble model was superior to that of the individual models, indicating that stacking may have significant implications for malaria disease prediction [8].

Despite the increasing volume of research utilising ML methods for malaria prediction and diagnosis, several significant gaps persist. First, numerous current studies depend on laboratory-based data [24] or microscopy images [25,26], which are frequently inaccessible in resource-limited environments. Second, although individual ML models have demonstrated encouraging outcomes in malaria predictions [27], there has been insufficient focus on the systematic assessment of ensemble learning approaches for malaria diagnosis utilising routine clinical and demographic information. Third, the majority of existing research is geographically concentrated outside of Southern Africa [28,29,30,31,32,33], including studies in East Africa and Asia. Given differences in malaria transmission dynamics, healthcare infrastructure, and data availability, findings from these regions may not be directly generalizable to countries such as Zimbabwe, where malaria continues to be endemic, and healthcare resources are limited [5,34,35].

In addressing these challenges, this paper aims to bridge the existing gap by developing and evaluating an ensemble ML framework for malaria diagnosis. While the concept of ensemble learning is well known and has been explored in other regions, the contribution of this work lies in its systematic evaluation within a real-world, resource-limited Southern African context. Furthermore, although symptom-based predictors for malaria are well recognised, their structured application and validation within an advanced machine learning framework designed for resource-limited settings signify a meaningful contribution.

This paper examines a variety of individual machine learning models, which include Logistic Regression (LR), Random Forest (RF), Decision Trees (DT), Gradient Boosting (GB), K-Nearest Neighbours (KNN), Naive Bayes (NB), and XGBoost, alongside ensemble models including Bagging, Stacking, Soft Voting, and AdaBoost, within the scope of malaria diagnosis using clinical and demographic data from Zimbabwe. This systematic comparison, particularly in a Sub-Saharan African context, is a key contribution, as most existing research is geographically concentrated in West Africa [28], East Africa [3,21,22], and Asia [8,25]. By focusing on readily available clinical and demographic data, the study offers a practical and scalable solution where more complex diagnostics are unavailable or prohibitively expensive.

## 2. Materials and Methods

### 2.1. Study Design and Data Collection

**Figure 1:**
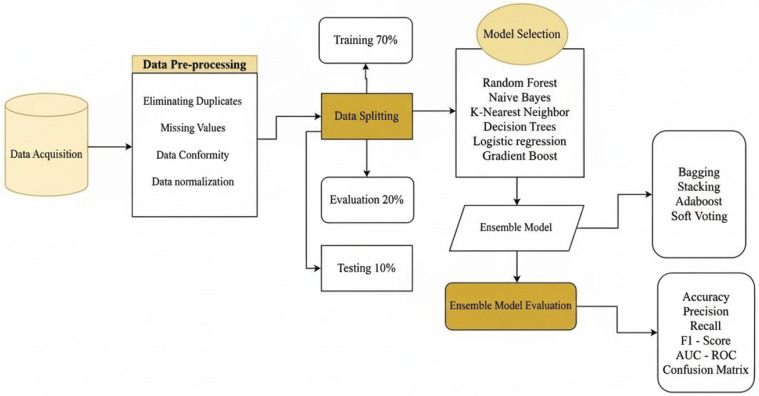
Methodology framework summarising the various steps of the study from data acquisition up to model evaluation.

This paper followed the methodology described in Figure 1. A retrospective quantitative study was conducted using anonymised patient records from Gweru Provincial Hospital (urban setting) and Gutu Mission Hospital (rural setting) in Zimbabwe, selected to represent high and low malaria transmission areas. Data were collected from routinely maintained hospital records, including outpatient registers and laboratory records. Information was manually extracted from these sources, as electronic health record systems were not available. A structured data collection form was developed in Microsoft Excel (Version 365, Microsoft Corporation, Redmond, WA, USA) to ensure consistent capture of variables. The extracted variables included demographic characteristics and clinical features, along with confirmed malaria test results. To ensure data accuracy, a two-step verification process was implemented, whereby the extracted data were cross-checked against the original outpatient registers and corresponding entries in the Excel dataset.

### 2.2. Inclusion and Exclusion Criteria

Patient records from January 2022 to December 2024 with a confirmed malaria diagnosis (positive/negative) with comprehensive clinical and demographic data were included in this study. However, records with missing key variables, such as malaria test results, cases with co-infections such as typhoid, or ambiguous diagnostic outcomes, were excluded from the study.

### 2.3. Sample Size and Sampling Procedure

This paper used the entire dataset of eligible records, 637 participants tested for malaria with complete details from Gutu Mission Hospital and Gweru Provincial Hospital. The dataset consisted of participants who tested either positive or negative for malaria.

### 2.4. Dependent and Independent Variables

The outcome variable for this study, referred to as malaria status, is a binary variable that captures whether the patient was positive or negative for malaria. The predictors included clinical symptoms (fever, chills, headache, abdominal pain, diarrhea (binary variables)) and demographic features (age, gender, residence and travel history).

### 2.5. Data Preprocessing

A Microsoft Excel file was created for the malaria dataset to facilitate data pre-processing in R version 4.4.1 (R Foundation for Statistical Computing, Vienna, Austria) [36]. Data pre-processing in this paper comprised data cleaning, feature selection, class-imbalance handling, data encoding, and data splitting. To ensure reproducibility, detailed hyperparameter tuning and model configuration can be found in the R code link provided in the Appendix A.

### 2.6. Data Cleaning

Data entry typos, wrong numerical values and incorrect data formats were identified by comparing the paper-based medical records and the computed spreadsheet, and they were corrected to align with the original dataset. Duplicate records were checked for by verifying key identifiers such as patient ID and test date, and were removed to avoid redundant data from skewing the ML models. The dataset was then checked for outliers to make sure no extreme values might affect predictive accuracy. Data points with extreme values were capped, which means replacing extreme values with upper and lower threshold values.

### 2.7. Feature Selection

Variance inflation factor (VIF) and Pairwise Pearson correlation coefficients (r) were used to study the relationships between the variables. Recursive Feature Elimination (RFE) guided by RF was used for feature selection, choosing the most effective features for model building.

### 2.8. Handling Class Imbalance

An imbalanced dataset is when one class outnumbers the other [37]. This imbalance can lead to biases towards the majority class [38]. The majority of the malaria diagnosis results from the collected data were negative (562) compared to the 75 positive cases, very underrepresented in a study comprising 637 participants. Synthetic Minority Oversampling Technique (SMOTE) was used to address data imbalance using the SMOTE function in R. This was done through creating synthetic samples for the minority class by interpolating between existing minority class [39,40].

### 2.9. Data Encoding

One-Hot encoding was used to change categorical variables into numerical representations to make them compatible with ML algorithms. Age was grouped into categorical bins (0–5 years, 6–15 years, 16–30 years, 31–45 years, 46–60 and 61+ years) (Table 1). The decision to categorise age was based on clinical and epidemiological literature, which has successfully proven that working with age bins for malaria-related studies takes into consideration that malaria incidence and severity vary non-linearly across age groups and effectively improves model performance [41,42,43].

### 2.10. Data Splitting

The dataset was split using stratified sampling into training, evaluation and testing sets at a ratio of 70:20:10, respectively. The 70% for training allows for sufficient model training, and the 20% for evaluation ensures there is enough data for hyperparameter tuning and evaluation of model performance. The 10% for testing was used for the final evaluation of model performance and was strictly unseen during training and hyperparameter tuning to provide an unbiased estimate for the model’s performance on unseen data.

### 2.11. Individual Model Selection and Training

Seven individual ML classification algorithms were selected and trained to evaluate their contributions to specific ensemble methods. The models included LR, GB, KNN, XGBoost, RF, DT, and NB. Model selection was guided by a comprehensive review of the literature on malaria status prediction [3,21,44]. All seven models were trained using 70% of both the balanced and unbalanced datasets to compare their predictive performance. Detailed descriptions and equations can be found in Appendix A.

### 2.12. Hyperparameter Tuning

Optimisation of model performance was done by tuning hyperparameters relevant to specific ML classifiers. Lasso regression was used to prevent overfitting and improve the generalisation of LR. LR was also trained on a 5-fold cross-validation to improve performance.

RF and XGBoost were tuned using Grid Search. The hyperparameter mtry in RF was tested for values c(1,2,3,4) and 10-fold cross-validation was applied to verify for favourable parameter settings. For XGBoost, the hyperparameters tuned included the number of trees, learning rate and maximum depth. 5-fold cross-validation was used to evaluate the most favourable hyperparameters for XGBoost.

Cross-validation-based pruning was used for DT. It was applied to optimise the tree’s complexity and prevent overfitting using the rpart function in R. Random search was used for tuning hyperparameters for KNN, where tune length was set to 10 to allow KNN to explore a range of values for *k*. For GB, Bayesian optimisation was used to tune the hyperparameters such as the number of trees, learning rate and tree dept. Cross-validation was used for NB, and tuneLength was set to 10.

### 2.13. Ensemble Model Selection and Building

Using ensemble techniques through the integration of various ML classification algorithms has proven to achieve greater precision performance compared to utilising a solitary technique [45,46]. The study employed bagging, stacking, soft voting and adaboost.

Bagging works by reducing variance and preventing overfitting by training multiple models on different subsets of the training data and averaging their predictions [47,48]. RF was preferred for the bagging method as it uses multiple decision trees during training [49]. Training of the bagging model was done using the randomForest function in R.

Stacking uses basic-level meta classifiers and amalgamates them with meta-learner classifiers [48,50]. The base learners used for stacking are RF,DT,KNN,GB,NB and XGBoost and LR as the base classifier. Prediction models were first obtained from each of the base models. A new dataset (predictions_stacked) was created, and it contained all base learners and the actual target variable (Diagnosis).

Soft Voting used LR, RF, DT, KNN, GB, NB and XGBoost as base models. These base models were averaged, and the predicted probabilities were combined [51]. AdaBoost used the ada package in R, with 100 iterations per iteration. AdaBoost adjusts the weights of misclassified instances to improve the performance of weak learners [50,52]. Mathematical formulations of these models are provided in Appendix A.

### 2.14. Individual and Ensemble Model Performance Evaluation

The individual and ensemble models were evaluated on their performance in malaria prediction using metrics such as accuracy, recall, precision, confusion matrix, F1 score and AUC-ROC. Predictions and predicted probabilities were generated for each model, and the performance metrics were calculated and compared. For ensemble models, the same metrics were used to assess the effectiveness. Model performance was also compared between individual and ensemble models.

### 2.15. Ethical Review

Ethical clearance was acquired from the Research Ethics Committee of Gweru Provincial Hospital (Approval Code: 26092024/040; Approved on 26 September 2024).For Gutu Rural District Hospital, formal ethical approval was issued through the administrative endorsement by the hospital authority. Measures were implemented to ensure any ethical issues relating to the study were addressed. All patient identifiers, such as names, national identification numbers, and contact details, were removed during data collection. For issues related to data security, the dataset was kept secure and accessible to authorised people only.

## 3. Results

### 3.1. Analysis of Demographic and Clinical Variables Associated with Malaria Diagnosis

A total of 637 participants with an age range of 1 to 90 years were included in this paper. The mean age was 30.7 ± 21.4 years, indicating a wide variation in participant ages. Out of the 637 participants 49.9% were from Gweru Provincial Hospital while 50.1% were from Gutu Mission Hospital. The distribution of gender was similar between groups, with no significant difference observed in their diagnosis results (p=0.483, Table 2).

However, participants who tested positive were significantly more likely to report fever (84.0% vs. 66.0%,p= 0.002), chills (81.3% vs. 62.3%,p=0.001), diarrhea (41.3% vs. 26.5%,p=0.007), and abdominal pain (50.7% vs. 29.5%,p<0.001) compared to those who tested negative (Table 2). A higher proportion of positive cases had a recent travel history (49.3% vs. 35.2%,p=0.017) and resided in rural areas (65.3% vs. 51.1%,p=0.020). No significant differences were observed in the distribution of headache or age groups between the two diagnosis categories (Table 2).

### 3.2. Results of Feature Selection

**Figure 2:**
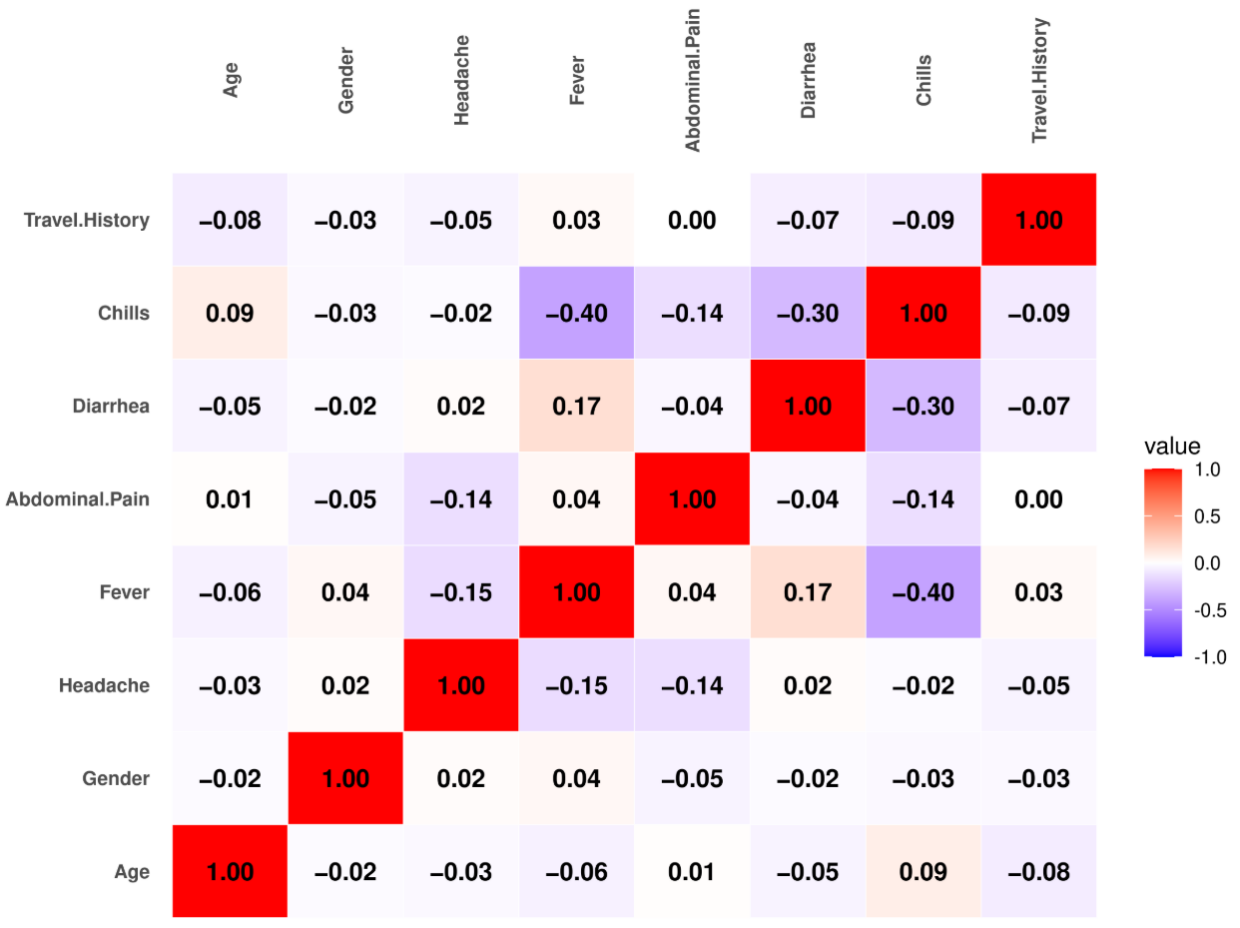
Feature Correlation Matrix for Malaria Predictors.

A correlation analysis of selected numeric and binary-encoded variables (age, gender, headache, fever, abdominal pain, diarrhoea, chills and travel history) confirmed that the predictors are relatively independent of each other. The pairwise Pearson correlation coefficients (r) (Figure 2) show that most of the correlations are close to zero, indicating weak linear relationships between variables. The highest observed correlation was between chills and fever, with a coefficient of −0.40. Travel history showed weak to negligible correlations with all other features, except a moderate negative correlation with age (r=−0.08).

**Figure 3:**
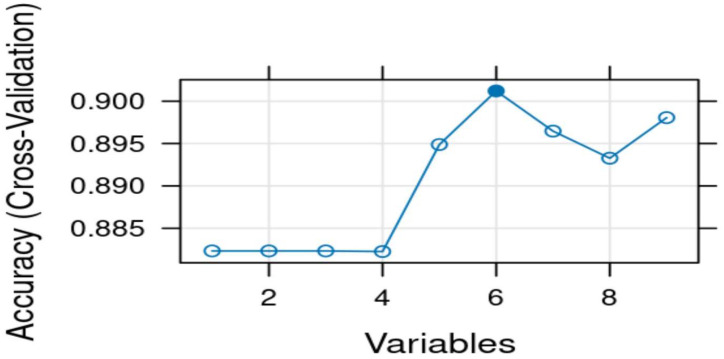
Feature Importance Analysis with Recursive Feature Elimination.

Using RFE, the top five variables identified (Figure 3) were chills, fever, diarrhea, travel history, and abdominal pain. All variables demonstrated statistical significance based on chi-square tests (Table 2), with *p*-values of 0.0019 for chills, 0.003 for fever, 0.01 for diarrhea, 0.02 for travel history, and 0.0004 for abdominal pain.

VIF values for the predictors were all below 5, indicating no significant collinearity among predictors (Table 3). The highest VIF was 1.35 for chills, and the lowest VIF was 1.01 for gender. Findings align with the weak correlations observed in the correlation matrix (Figure 2).

**Figure 4:**
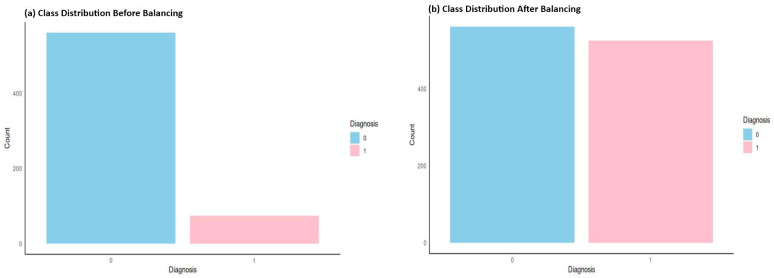
Class distribution before (**a**) and after (**b**) balancing with SMOTE.

SMOTE successfully addressed the data imbalance issue to improve model performance [Figure 4].

### 3.3. Machine Learning

**Figure 5:**
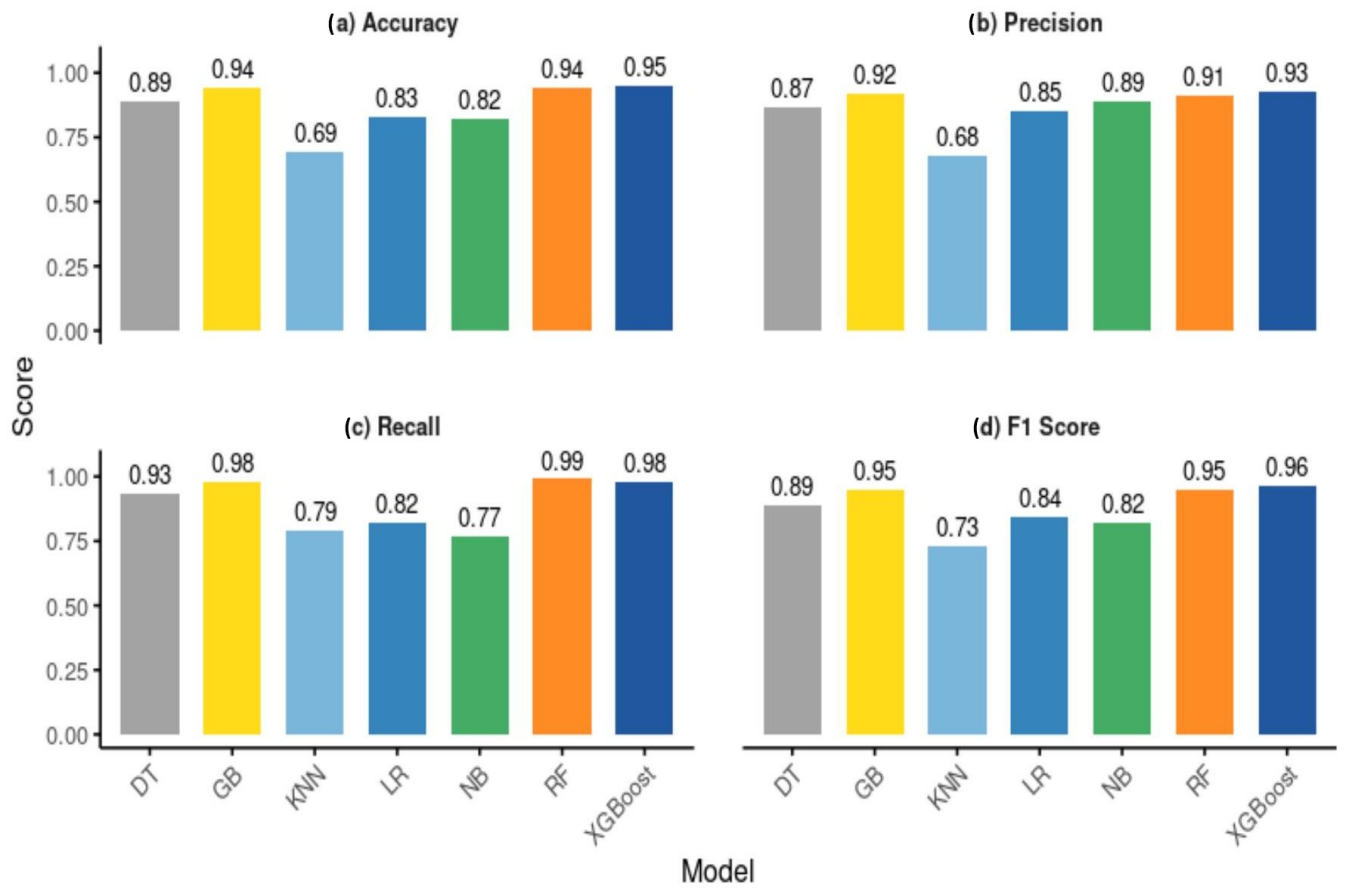
Model Performance Evaluation on Test set: (**a**) Accuracy, (**b**) Precision, (**c**) Recall, and (**d**) F1-score.

The performance of the seven ML individual classifiers (LR, RF, DT, GB, KNN, NB and XGBoost) were evaluated using six metrics. XGBoost got the highest accuracy (0.95). GB and RF both got an accuracy of 0.94. DT achieved an accuracy of 0.89, while LR had 0.83 and NB had 0.82, showing lower but comparable performance. KNN had the lowest accuracy of 0.69. XGBoost achieved the highest precision (0.93). RF and GB had precision values of 0.91 and 0.92, respectively. DT (0.87) and LR (0.85) achieved moderate precision values. KNN had the lowest precision of 0.68. Recall was strongest for RF at 0.99. XGBoost and GB both got a recall of 0.98. DT obtained 0.93, showing a good recall performance. LR (0.82) and NB (0.77) demonstrated more limited sensitivity. KNN achieved 0.79 recall, performing better in this metric than in others (Figure 5).

**Figure 6:**
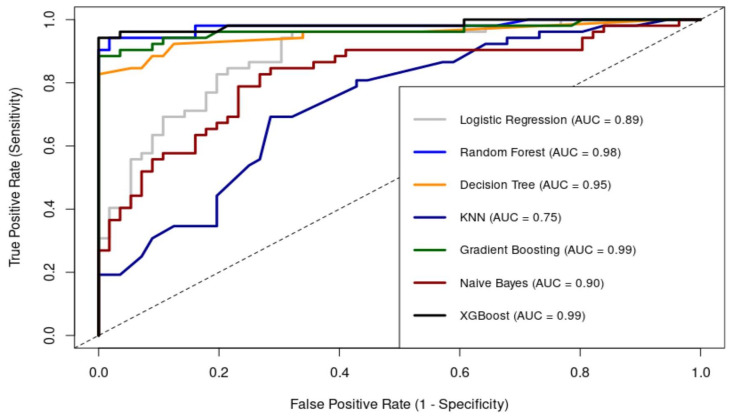
Receiver Operating Characteristic Curve Analysis on test set Performance.

The AUC-ROC values, indicating overall classification ability across all thresholds, were strongest for XGBoost and GB (both 0.99). RF achieved 0.98, DT (0.95), and NB (0.90), while LR achieved 0.89. KNN (0.75) had the lowest AUC-ROC performance (Figure 6).

**Figure 7:**
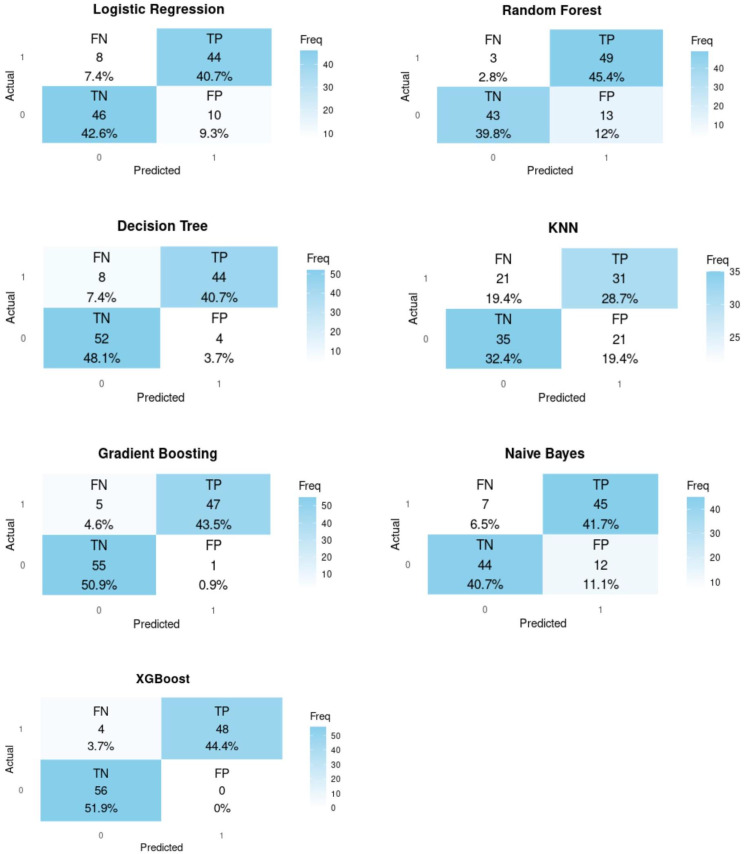
Confusion Matrix Performance for Models on Test set (1 = positive class, 0 = negative class).

Confusion matrices were used to evaluate the models, where each matrix shows the number of actual malaria cases (positives) and non-cases (negatives) correctly or incorrectly predicted by the model (Figure 7). LR correctly classified 40.7% true positives (TP) and 42.6% true negatives (TN), then misclassified 7.4% of actual positives as false negatives (FN) and 9.3% of actual negatives as false positives (FP). XGBoost achieved strong performance, with 44.4%TP and 51.9%TN. Impressively, it recorded 3.7%FN and 0%FP, making it the model with the lowest error. GB correctly identified 43.5% of TP and 50.9% of TN. It had minimal misclassifications of 4.6%FN and only 0.9%FP.

**Figure 8:**
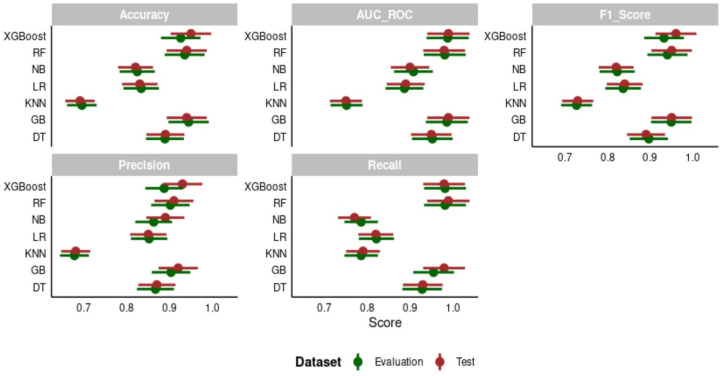
Comparison of Model Performance on Evaluation and Test Sets.

Comparative performance analysis of models between the test and evaluation sets showed consistent patterns in predictive accuracy and reliability. XGBoost showed superior performance across all metrics, achieving the highest accuracy (0.95 test, 0.93 eval), F1-score (0.96 test, 0.93 eval), AUC-ROC (0.99), precision (0.93) and recall (0.98). RF and GB models had a nearly similar performance to XGBoost. DT showed moderate performance with accuracy and F1-scores around 0.89 (Figure 8).

**Figure 9:**
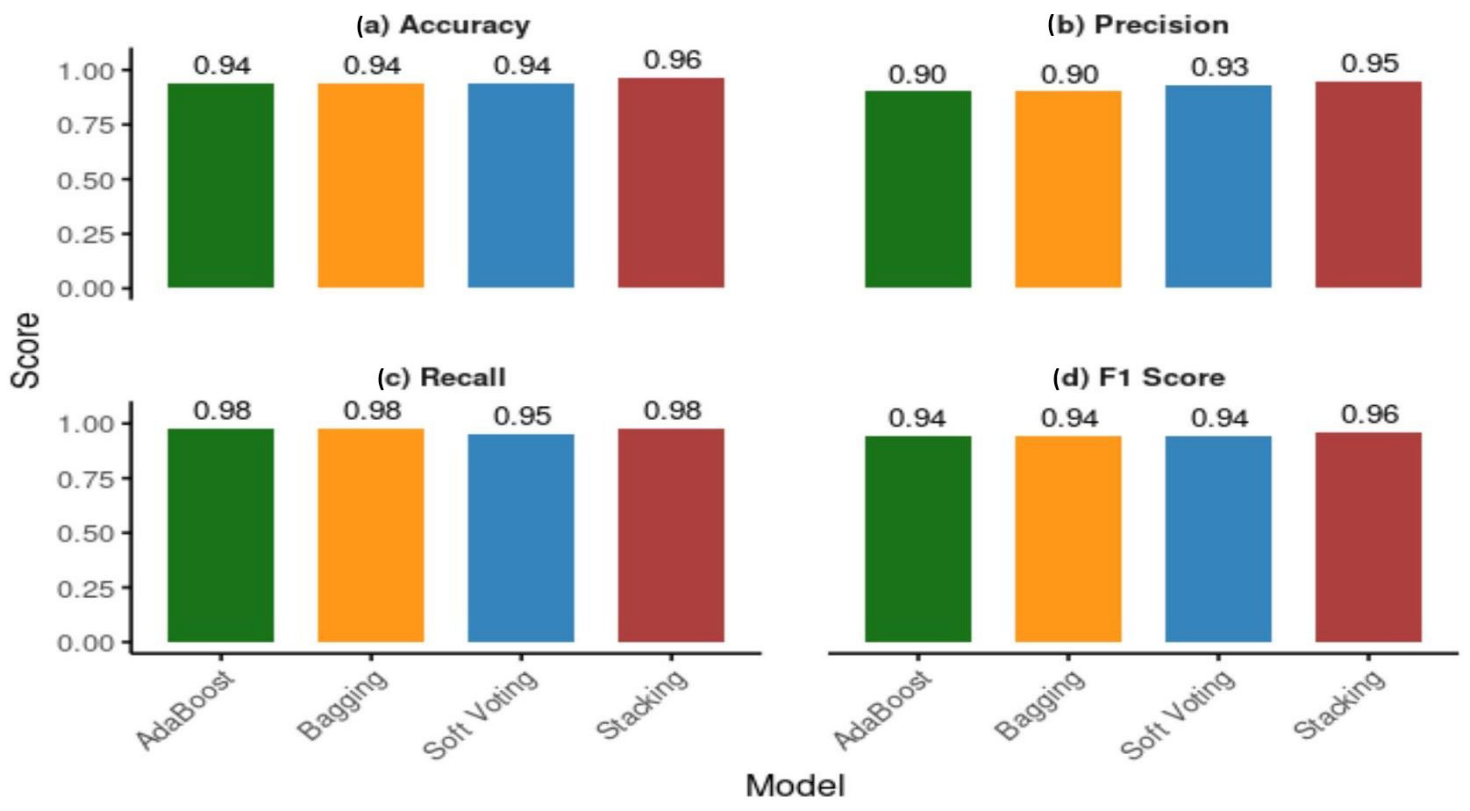
Ensemble Model Performance on test set: (**a**) Accuracy, (**b**) Precision, (**c**) Recall, and (**d**) F1-score.

The Stacking model demonstrated the highest overall performance across all evaluation metrics. Stacking has an accuracy of 96% while Soft Voting, Bagging and AdaBoost classifiers achieved an accuracy score of 94%. Soft Voting had a slightly higher precision (93%). Stacking had the highest precision of 95% (Figure 9).

**Figure 10:**
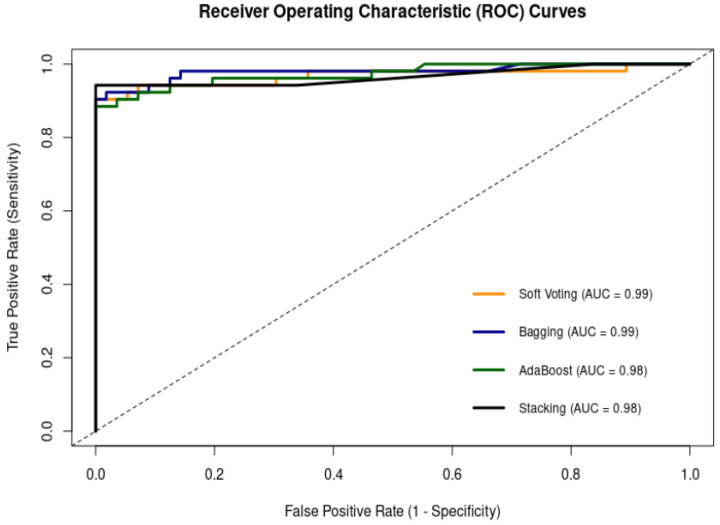
ROC Curve Analysis on test set Performance for Ensemble Models.

The ROC curves showed exceptional discriminative performance across all ensemble models. Soft Voting and Bagging achieved a high classification capability (AUC =0.99). AdaBoost and Stacking had AUC values of 0.98. All ensemble models maintained high sensitivity with their curves hugging the top-left corner of the plot, a nature only high-quality classifiers take. The clustering of AUC scores between 0.98 and 0.99 confirms that all ensemble methods provide clinically reliable diagnostic performance (Figure 10).

**Figure 11:**
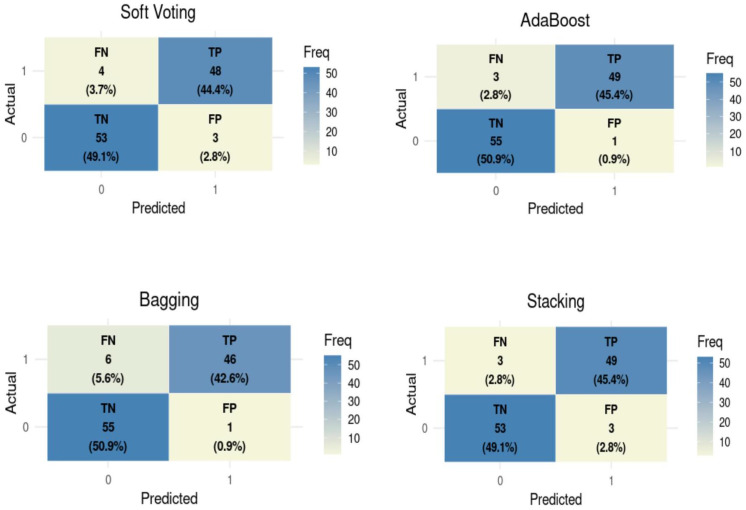
Confusion matrix performance of ensemble models on the test set (1 = positive class, 0 = negative class).

Confusion matrices for the four ensemble models show distinct performance quality in malaria classification (Figure 11). Soft Voting correctly identified 48 TP cases (44.9%) and 53 TN (49.1%), with error rates 4 FN (3.7%) and 3 FP (2.8%). Bagging had stronger specificity, with only 1 FP and 55 TN, with 6 FN at 5.6%. AdaBoost demonstrated the best sensitivity among all ensemble models, with 3 FN (2.8%) and 49 TP (45.4%) and only 1 FP. Stacking matched AdaBoost’s sensitivity with 3 FN at 2.8%, 49 TP at 45.4%, but with more FP. All models maintained FP rates below 3%.

## 4. Discussion

The findings of this research demonstrate the potential impact of ML ensemble models in enhancing malaria diagnosis, particularly in developing countries where there is poor accessibility and insufficient diagnostic tools for malaria. The results show that the Stacking ensemble model outperformed all individual models, obtaining an accuracy of 0.96, precision =0.95, recall =0.98, F1 score =0.96 and AUC-ROC =0.98. Of the 637 participants from Gweru Provincial Hospital (49.9%) and Gutu Mission Hospital (50.1%), a proportion of 50.9 were males and 49.1% were females. The age of the participants had a mean of 31±21 years. A total of 75 cases were positive, with 54.7% of the 75 cases being males and 45.3% females showing a slightly higher prevalence in males, although the difference did not attribute any statistical significance (p=0.56).

The findings highlight that Travel history (p=0.02) is a significant demographic predictor of malaria incidence, suggesting that travellers may experience different risk exposures and prevention behaviours compared to others. Clinical symptoms such as chills, fever, abdominal pain and diarrhea were significant predictors of malaria (p<0.05). This reinforces the clinical utility of these readily observable symptoms as diagnostic markers, especially in settings where rapid diagnostic tests (mRDTs) may have reduced sensitivity at low parasite densities or produce false results.

These results align with existing literature, which emphasises how physical clinical symptoms of malaria can be relied on for diagnosis, especially in malaria endemic areas [53]. Some studies argue that demographic factors are as important as clinical symptoms, reporting gender and age as having a huge impact on malaria incidence [54,55]. Other literature reports that while demographics are found to be related to malaria incidence, different geographical and socio-economic conditions impact their significance to malaria epidemiology [56,57]. Early diagnosis of malaria through timely identification of clinical symptoms can potentially improve malaria management and control in endemic localities [54,58].

From a modelling perspective, the performance evaluation of the ML models, measured by considering all the metrics, showed that ensemble models outperform individual ML models. XGBoost achieved an accuracy of 0.95 while RF and GB got 0.94, indicating strong generalizability. A comparison of the individual models’ performance on the evaluation and test datasets (Figure 8) showed consistent performance, proving that there was minimal overfitting due to effective hyperparameter tuning. Models such as KNN and NB, which demonstrated moderate model performance, could suggest their applicability for specific scenarios with fewer complexities.

Beyond predictive performance, while traditional diagnostic methods remain standard, they require trained personnel and consistent supply chains, and in some cases, laboratory infrastructure [59]. ML-based approaches that use routinely collected clinical data may offer a low-cost decision support tool, aiding in the timely diagnosis of high-risk patients, particularly in settings where resources are limited. In facilities where mRDTs are in short supply, such models could assist preliminary risk stratification using readily available patient information. ML-based models can be deployed with minimal additional costs, especially when integrated into mobile health platforms [60]. However, initial implementation costs, including system development, validation and training of workers, should be considered.

While all the ML ensemble models achieved high diagnostic performance (Figure 9), the Stacking ensemble model outperformed the other ensemble models, obtaining a high malaria diagnostic performance across all metrics. Bagging, Stacking and AdaBoost achieved 0.98 recall, and Soft Voting got 0.95. This superior performance is clinically relevant because it means more reliable identification of malaria cases, which is critical for timely treatment and preventing severe outcomes.

The results also align with studies of Mahajan et al. [61] and Rajab et al. [62], who obtained very high precision scores for Bagging, Stacking and AdaBoost, emphasising that ensemble models are reliable in boosting sensitivity in malaria diagnosis. F1 scores for the ensemble models were high, with Stacking and AdaBoost at 0.96. Soft Voting and Bagging got 0.94, indicating a strong overall performance. The paper’s results for the F1 score of the ensemble model performance highlight the potential of the ensemble models at balancing precision and recall. Ensemble models demonstrated strong reliability in making predictions with minimal errors (Figure 11). The top-performing individual models in our study were XGBoost, GB and RF, while the top-performing ensemble model was stacking.

The performance achieved in this study is comparable to that reported in similar studies, particularly those utilising non-laboratory clinical data. Traditional models, including logistic regression, are effective for inference but are not optimised for predictive performance [19]. The greater accuracy of the stacking ensemble (0.96 compared to 0.83) exemplifies this difference. However, the performance of ensemble methods is dependent on the characteristics of the data and the diversity of the models involved [63]. In this analysis, the integration of various algorithms and SMOTE-based class balancing (75 positive cases versus 562 negative cases) improved performance by capturing non-linear relationships and utilising complementary strengths [3]. As such, ensemble methods are best regarded as supplementary tools for applications that emphasise prediction.

One limitation of this study was the relatively small number of malaria-positive cases in the dataset. The study relied on retrospectively collected clinical records from clinical facilities where patient information was primarily recorded manually. As a result, some records contained ambiguous entries, which limited the number of usable cases available for analysis. Despite the data constraints, the study demonstrates the feasibility of using clinical data for ML modelling in malaria diagnosis.

## 5. Conclusions

The paper highlights a significant gap in the literature regarding the development and systematic assessment of ensemble learning approaches for malaria diagnosis utilising routine clinical and demographic information in resource-limited settings. This study fills this gap by systematically comparing the performance of several individual ML classifiers with ensemble learning techniques. The strong performance of ensemble models, particularly the stacking approach, is consistent with previous studies that have shown improved predictive accuracy when combining multiple classifiers. However, unlike many prior studies that rely on laboratory or imaging data, this study highlights the feasibility of using readily available clinical information, making the approach more applicable in low-resource environments.

Key clinical features, including fever, chills, and abdominal pain, were identified as important predictors, reinforcing their relevance in symptom-based screening. These findings provide context-specific evidence for Zimbabwe, where limited research has explored the use of ensemble machine learning models for malaria diagnosis using real-world clinical data. Despite these contributions, limitations related to sample size, data completeness, and model generalisability remain. The proposed models require prospective validation and careful integration into clinical workflows. Future work should focus on large-scale validation and implementation within digital health platforms to assess usability, acceptance, and real-world impact.

## Figures and Tables

**Table 1:** Data Coding Description.

Feature Name	Feature Description	DataType	Levels	Encoding
Residence	Living Environment	Categorical	Gutu = 1, Gweru = 2	1 and 2
Age	Patient’s age in years	Integer	0 to 95	Continuous (Binning)
Gender	Biological sex of a patient	Categorical	Male =1, Female =0	1 and 0
Headache	Presence of headache symptom	Binary Integer	Yes =1, No =0	1 and 0
Fever	Presence of fever symptom	Binary Integer	Yes =1, No =0	1 and 0
Abdominal Pain	Presence of abdominal Pain symptom	Binary Integer	Yes =1, No =0	1 and 0
Diarrhea	Presence of diarrhea symptom	Binary Integer	Yes =1, No =0	1 and 0
Chills	Sudden cold sensations	Binary Integer	Yes =1, No =0	1 and 0
Travel History	Recent travel to malaria-endemic areas	Binary Integer	Yes =1, No =0	1 and 0
Diagnosis	Malaria diagnosis outcome	Categorical	Positive =1, Negative =0	1 and 0

**Table 2:** Baseline characteristics for all the participants in this study.

Variable	Category	Negative (n=562)	Positive (n=75)	Total (n=637)	*p*-Value
Gender	Male	283 (50.4%)	41 (54.7%)	324 (50.9%)	0.483
	Female	279 (49.6%)	34 (45.3%)	313 (49.1%)	
Fever	Yes	371 (66.0%)	63 (84.0%)	434 (68.1%)	0.002 **
	No	191 (34.0%)	12 (16.0%)	203 (31.9%)	
Chills	Yes	350 (62.3%)	61 (81.3%)	411 (64.5%)	0.001 **
	No	212 (37.7%)	14 (18.7%)	226 (35.5%)	
Headache	Yes	521 (92.7%)	69 (92.0%)	590 (92.6%)	0.826
	No	41 (7.3%)	6 (8.0%)	47 (7.4%)	
Diarrhea	Yes	149 (26.5%)	31 (41.3%)	180 (28.3%)	0.007 **
	No	413 (73.5%)	44 (58.7%)	457 (71.7%)	
Abdominal Pain	Yes	166 (29.5%)	38 (50.7%)	204 (32.0%)	<0.001 **
	No	396 (70.5%)	37 (49.3%)	433 (68.0%)	
Travel History	Yes	198 (35.2%)	37 (49.3%)	235 (36.9%)	0.017 *
	No	364 (64.8%)	38 (50.7%)	402 (63.1%)	
Location	Rural	287 (51.1%)	49 (65.3%)	336 (52.7%)	0.020 *
	Urban	275 (48.9%)	26 (34.7%)	301 (47.3%)	
Age Group	0–5	92 (16.4%)	8 (10.7%)	100 (15.7%)	0.298
	6–15	62 (11.0%)	6 (8.0%)	68 (10.7%)	
	16–30	160 (28.5%)	19 (25.3%)	179 (28.1%)	
	31–45	104 (18.5%)	21 (28.0%)	125 (19.6%)	
	46–60	89 (15.8%)	11 (14.7%)	100 (15.7%)	
	>60	55 (9.8%)	10 (13.3%)	65 (10.2%)	

Percentages shown are column percentages (within diagnosis groups). Significant *p*-values are indicated by * (<0.05), ** (<0.01).

**Table 3:** VIF analysis output for correlation analysis.

Variable	VIF Value
Chills	1.35
Fever	1.24
Diarrhoea	1.12
Headache	1.06
Abdominal Pain	1.06
Residence	1.04
Travel History	1.04
Age	1.03
Gender	1.01

## Data Availability

The datasets used and/or analyzed during the current study are available from the corresponding author on reasonable request. The codes used for this study are publicly available at https://github.com/panawaShe/malaria-ml-diagnosis, accessed on 12 March 2026.

## Abbreviations

The following abbreviations are used in this manuscript:

ML	Machine Learning
AI	Artificial Intelligence
LR	Logistic Regression
RF	Random Forest
DT	Decision Trees
GB	Gradient Boosting
KNN	K-Nearest Neighbors
NB	Naive Bayes
AUC-ROC	Area Under the Receiver Operating Characteristic Curve
SMOTE	Synthetic Minority Oversampling Technique
mRDTs	malaria Rapid Diagnostic Tests
ARIMA	Autoregressive Integrated Moving Average
STL+ARIMA	Seasonal and Trend Decomposition using Loess
BP-ANN	Back Propagation Artificial Neural Network
LSTM	Long Short-Term Memory

## Appendix A. Mathematical Formulation of Machine Learning Models

### Appendix A.1. Logistic Regression (LR)

LR is a supervised algorithm used for binary classification. It takes input as independent variables and produces a probability between 0 and 1 [64]. In this study, LR was applied to estimate the probability of malaria infection (positive or negative) based on clinical and demographic features. The equation used is as follows:

$$P\left(Y=1|X\right)=\frac{1}{1+e^{-\left(\beta_{0}+\beta_{1}\cdot \;\mathrm{Age}\;+\beta_{2}\cdot \;\mathrm{Temperature}\;+\beta_{3}\cdot \;\mathrm{Fever}\;+\ldots \right)}}$$

where $\beta_{0}$ is the intercept and $\beta_{i}$ are the coefficients associated with each predictor variable. *Y* represents malaria status (1= positive, 0= negative ), and X denotes the set of clinical and demographic features. The model outputs a probability between 0 and 1, indicating the likelihood of malaria positivity.

### Appendix A.2. Decision Trees (DT)

DTs were used to classify individuals as malaria positive or malaria negative based on clinical and demographic variables. The model works by repeatedly splitting the dataset into smaller groups using variables, with the aim of improving the separation between positive and negative cases.

At each step, the algorithm selects the variable and threshold (e.g., temperature > 37.5 °C) that best separates the outcome. This process continues until no further meaningful splits can be made. At the final nodes, the predicted class is determined by the majority class among observations in that group:

$${\hat{y}}_{m}={\mathrm{argmax}}_{k}\sum_{i}I\left(y_{i}=k\right),\;\mathrm{for}\;i\in R_{m}$$

where $R_{m}$ represents the subset of patients in node *m*, and I(·) is an indicator function. *k* represents the class label (malaria positive or negative). The quality of each split is evaluated using the Gini impurity:

$$\mathrm{Gini}\left(Q\right)=1-\sum_{k}{p_{k}}^{2}$$

where $p_{k}$ is the proportion of observations belonging to class *k* in node *Q*. The algorithm selects splits that minimize the Gini impurity, resulting in nodes that are more homogeneous in terms of class distribution [65].

### Appendix A.3. Random Forest (RF)

RF is an ensemble learning method that builds multiple DTs using the same clinical and demographic variables and combines their predictions to improve classification performance. Instead of relying on a single tree, RF aggregates the results from many trees to produce a more stable and accurate prediction of malaria status. Each tree is trained on a different random subset of the dataset, and at each split, a random subset of predictor variables is considered. The final prediction is determined by majority voting across all trees:

$$\hat{y}=\mathrm{mode}\left(T_{1}\left(x\right),T_{2}\left(x\right),\ldots ,T_{B}\left(x\right)\right)$$

where $T_{b}\left(x\right)$ represents the prediction of the *b*-th decision tree, and *B* is the total number of trees in the forest [65].

### Appendix A.4. K-Nearest Neighbours (KNN)

KNN algorithm classifies individuals based on the similarity of their clinical and demographic characteristics to those of other patients in the dataset [66]. In this study, features such as age, temperature, and reported symptoms were used to determine similarity between cases. For a new patient, the algorithm identifies the *k* most similar individuals (nearest neighbours) in the dataset using a distance measure, typically Euclidean distance:

$$d\left(x,x_{i}\right)=\sqrt{\sum_{j=1}^{p}{\left(x_{j}-x_{ij}\right)}^{2}}$$

where *x* represents the feature vector of the new patient, $x_{i}$ represents the feature vector of an existing patient, and *p* is the number of features.

The predicted malaria status is then determined by the majority class among the *k* nearest neighbours:

$$\hat{y}={\mathrm{argmax}}_{c}\sum_{i\in N_{k}}I\left(y_{i}=c\right)$$

where $N_{k}$ denotes the set of the *k* nearest neighbours, I(·) is the indicator function, and *c* represents the class label (malaria positive or negative).

### Appendix A.5. Gradient Boosting (GB)

GB builds models sequentially, where each model corrects the errors of the previous one. In this study, GB allows improved prediction of malaria status by iteratively refining model performance.

$$F_{m}\left(X\right)=F_{m-1}\left(X\right)+\gamma_{m}h_{m}\left(X\right)$$

where $h_{m}\left(X\right)$ is the base learner fitted to the residual errors at iteration *m*, and $\gamma_{m}$ is the learning rate that controls the contribution of each new model [65].

### Appendix A.6. Naive Bayes (NB)

NB is a probabilistic classifier based on Bayes’ theorem, assuming that features are conditionally independent given the class label. In this study, NB was used to predict malaria status by calculating the probability of malaria infection given the presence of clinical symptoms and demographic characteristics. The model computes the posterior probability using:

$$P\left(\mathrm{Malaria}=1|X\right)=\frac{P\left(\mathrm{Malaria}=1\right)\prod_{j=1}^{p}P\left(x_{j}|\mathrm{Malaria}=1\right)}{P(X)}$$

where P(Malaria=1) is the prior probability of malaria in the dataset, $P(x_{j}|\mathrm{Malaria}=1)$ is the conditional probability of observing feature $x_{j}$ (e.g., fever, chills, travel history) given that the patient has malaria, and P(X) is the marginal likelihood (evidence) of the feature vector [67].

### Appendix A.7. Extreme Gradient Boosting (XGBoost)

XGBoost is an optimized implementation of GB that combines multiple weak learners to create a strong predictive model. In this study, XGBoost was used to classify malaria status by iteratively adding trees that correct errors from previous trees. The objective function at step *t* is:

$$L^{\left(t\right)}=\sum_{i=1}^{n}L\left(y_{i},{\hat{y}}_{i}^{\left(t-1\right)}+f_{t}\left(x_{i}\right)\right)+\Omega \left(f_{t}\right)$$

where *L* is the loss function, $y_{i}$ is the true malaria status, ${\hat{y}}_{i}^{\left(t-1\right)}$ is the prediction from the previous t−1 trees, $f_{t}$ is the *t*-th tree added to the model, and $\Omega (f_{t})$ is a regularization term that penalizes model complexity to prevent overfitting [68].

### Appendix A.8. Bagging (Bootstrap Aggregating)

Bagging (Bootstrap Aggregating) is an ensemble technique that reduces variance by training multiple models on different bootstrap samples of the dataset and aggregating their predictions. In this study, bagging was implemented using decision trees as base learners.

Given a dataset *D* with *N* samples, the algorithm proceeds as follows:

For b=1 to *B*:

1. Generate a bootstrap sample $D_{b}$ by randomly sampling *N* instances from *D* with replacement.
2. Train a decision tree $f_{b}$ on $D_{b}$ without pruning.

The final prediction for a new patient is determined by majority voting across all *B* models:

$$\hat{y}=\mathrm{mode}\left(f_{1}\left(x\right),f_{2}\left(x\right),\ldots ,f_{B}\left(x\right)\right)$$

where $f_{b}\left(x\right)$ represents the prediction (malaria positive or negative) from the *b*-th model [69].

### Appendix A.9. Stacking

Stacking is an ensemble learning technique that combines predictions from multiple diverse base learners using a meta-learner to produce a final prediction. In this study, six base learners (Random Forest, Decision Tree, K-Nearest Neighbours, Gradient Boosting, Naive Bayes, and XGBoost) were trained, and Logistic Regression (LR) was used as the meta-learner [70].

The stacking framework consists of two levels:

Level 1 (Base Learners): Each base learner $f_{j}$, for j=1,…,6, is trained on the training data. For each patient *i*, the base learners generate predictions which are combined to form a new feature vector:

$$z_{i}=\left[f_{1}\left(x_{i}\right),f_{2}\left(x_{i}\right),f_{3}\left(x_{i}\right),f_{4}\left(x_{i}\right),f_{5}\left(x_{i}\right),f_{6}\left(x_{i}\right)\right]$$

where $f_{1},\ldots ,f_{6}$ correspond to Random Forest, Decision Tree, K-Nearest Neighbours, Gradient Boosting, Naive Bayes, and XGBoost, respectively.

Level 2 (Meta-Learner): A meta-learner *F* (Logistic Regression) is trained on the new dataset $\left\{\left(z_{i},y_{i}\right)\right\}$, where $y_{i}$ is the true malaria status.

The final prediction for a new patient is given by:

$$\hat{y}=F\left(f_{1}\left(x\right),f_{2}\left(x\right),f_{3}\left(x\right),f_{4}\left(x\right),f_{5}\left(x\right),f_{6}\left(x\right)\right)$$

### Appendix A.10. Soft Voting

Soft Voting is an ensemble method that averages the predicted class probabilities from multiple classifiers and selects the class with the highest average probability [3]. In this study, soft voting combined predictions from LR, RF, DT, KNN, GB, NB, and XGBoost. For a given patient with feature vector *x*, each base classifier *ℓ*, where *ℓ* = 1,2,…,*L*, produces an estimated probability of malaria positivity:

$$P_{l}\left(Malaria=1|x\right)$$

The ensemble probability for malaria positivity is computed as the average of the individual classifier probabilities:

$$P_{ensemble}\left(Malaria=1|x\right)=\frac{1}{L}\sum_{l=1}^{L}P_{l}\left(Malaria=1|x\right)$$

The final predicted class is determined by selecting the class with the highest ensemble probability:

$$\hat{y}={\mathrm{argmax}}_{k\in \{0,1\}}P_{ensemble}\left(Malaria=k|x\right)$$

### Appendix A.11. AdaBoost

AdaBoost is an iterative boosting algorithm that combines multiple weak learners to create a strong classifier by assigning higher weights to misclassified instances [3]. In this study, AdaBoost was implemented with DTs as weak learners. The algorithm proceeds as follows:

Initialize weights for each observation:

$$D_{1}\left(i\right)=\frac{1}{N},\;i=1,2,\ldots ,N$$

For t=1,2,…,T:

1. Train a weak learner $h_{t}$ using the weighted dataset $D_{t}$.
2. Compute the weighted error:
  $$\varepsilon_{t}=\sum_{i=1}^{N}D_{t}\left(i\right)\;I\left(h_{t}\left(x_{i}\right)\neq y_{i}\right)$$
3. Compute the learner weight:
  $$\alpha_{t}=\frac{1}{2}\ln\left(\frac{1-\varepsilon_{t}}{\varepsilon_{t}}\right)$$
4. Update the weights:
  $$D_{t+1}\left(i\right)=\frac{D_{t}\left(i\right)\exp\left(-\alpha_{t}y_{i}h_{t}\left(x_{i}\right)\right)}{Z_{t}}$$
  where $Z_{t}$ is a normalization factor ensuring that:
  $$\sum_{i=1}^{N}D_{t+1}\left(i\right)=1$$

The final prediction is given by a weighted majority vote of the weak learners:

$$H\left(x\right)=\mathrm{sign}\left(\sum_{t=1}^{T}\alpha_{t}h_{t}\left(x\right)\right)$$

where $h_{t}\left(x\right)$ is the prediction of the *t*-th weak learner.
